# An overview of the new rifampicin-resistant tuberculosis regimens available at decentralised drug-resistant tuberculosis sites for persons older than six years

**DOI:** 10.4102/safp.v62i1.5092

**Published:** 2020-02-17

**Authors:** Madeleine Muller

**Affiliations:** 1 Nkqubela TB Hospital, Clinical, East London, South Africa; 2 Rural Doctors Association of Southern Africa, Johannesburg, South Africa

## Background

The rifampicin-resistant tuberculosis (RR-TB) programme has been subjected to dramatic and revolutionary advances over the past 5 years. ‘Rifampicin-resistant tuberculosis’ is used as a general term to encompass all the forms of RR-TB, including multidrug-resistant TB (MDR-TB) and extensively drug-resistant TB (XDR-TB). Through the development of new drugs and simpler, shorter and more acceptable treatment regimens, the success rate for both MDR-TB and XDR-TB has shown a dramatic improvement.

A policy supporting decentralised and deinstitutionalised drug-resistant TB (DR-TB) treatment provision at lower levels of the health system was introduced in 2011; however, largely because of complicated regimens, including daily injectables, implementation has varied. South Africa has been a global leader in this field and the work carried out in this country has affected global policy. It was one of the first countries to roll out the updated 2018 World Health Organization (WHO) recommendations to use an injection-free RR-TB regimen. In November 2019, the National Department of Health (NDOH) published a clinical reference guide for the management of RR-TB,^1^ giving in-depth guidance and protocols on all aspects of management of patients with RR-TB. The MDR-TB Policy Framework on Decentralised and Deinstitutionalised Management^2^ states that the majority of RR-TB care and services should be provided in the outpatient settings. This article highlights key aspects of the guideline for the primary care clinician managing patients in a decentralised RR-TB setting. Clear protocols and well-tolerated drugs make it possible to provide high-quality care to patients with uncomplicated MDR-TB in rural settings.

## Diagnosing drug-resistant tuberculosis

The entry point into the DR-TB programme is when the patient is confirmed as GeneXpert-positive, rifampicin resistant. The patient is classified as ‘RR-TB’, and notified and enrolled in a DR-TB programme. Note that DR-TB is always a laboratory diagnosis. The only exception to this rule is a child with TB symptoms and a close contact with DR-TB. If no sputum sample is acquired, the source case’s laboratory results would be used to design an appropriate regimen. Following the GeneXpert, the most important next investigation is the DR-TB reflex test.

### The drug-resistant tuberculosis reflex test

In South Africa, all patients with GeneXpert positive RR-TB must have a sputum sample sent for a DR-TB reflex test. This will automatically include the following:

1. *Smear microscopy for acid-fast bacilli* (*AFB*) to check for infectivity
2. *First-line Line Probe Assay* (*LPA*): For susceptibility to rifampicin (RIF) and isoniazid (INH) and to identify the two INH mutations (*inhA* and *katG*)
3. *Second-line LPA* (*SLPA*). For susceptibility to fluoroquinolones (FLQ) and injectables
4. *TB-culture*: All specimens will be cultured in case there were not enough bacilli to do the LPA test and for further phenotypic drug susceptibility testing (pDST)
5. *Phenotypic drug susceptibility testing*: Targeted depending on the LPA results:
  - If FLQ sensitive – will confirm susceptibility to levofloxacin (LFX) or moxifloxacin (MOX)
  - If FLQ-resistant – will confirm susceptibility to linezolid (LZD), bedaquiline (BDQ) and clofazamine (CFZ).

Line Probe Assay results are available within 8–18 days and make it possible to classify RR-TB and give appropriate treatment regimen.

### Medications available to treat drug-resistant tuberculosis

Drug-resistant tuberculosis has seen extraordinary progress in the development of effective medicines, including the development of new drugs (such as BDQ) and refashioning of known drugs (such as LZD and CFZ). The WHO currently defines three groups of drugs.

#### Group A drugs

This group contains three key medicines that, if the bacilli are susceptible to them, are always included in the regimen.

**Bedaquiline:** BDQ has transformed the DR-TB programme. Given three times a week as a standard 6-month course, it has dramatically increased the success rate in XDR since its introduction in 2014. It is relatively well tolerated, but because of a small risk of cardiac arrhythmia, it requires regular electrocardiogram (ECG) monitoring.

**Levofloxacin:** LFX has less Q wave–T wave (QT) interval effect than MOX and is therefore our drug of choice to accompany BDQ. The FLQ has been a key part of DR-TB regimens since MDR-TB was first identified.

**Linezolid:** It is a powerful drug and is key to all regimens. It has a risk of bone marrow suppression and full blood count (FBC) must be monitored on a monthly basis.

#### Group B drugs

This group contains two drugs that are excellent additions to a regimen.

**Terizidone:** TZD has been a part of XDR-TB regimen since the early days and is a key drug in our longer regimens. Adverse events include neuropsychiatric side effects and peripheral neuropathy.

**Clofazimine:** CFZ is well tolerated but sometimes causes the skin to acquire an orange hue in light-skinned patients and a darker hue in dark-skinned patients.

#### Group C drugs

Group C drugs are mostly used in rescue regimens or when Group A or B drugs are not tolerated.

Drugs with a lower efficacy include the first-line TB drugs: pyrazinamide (PZA), ethambutol (EMB) and high-dose INH (hINH).

New Group C drugs include delamanid (DLM), a recent addition to the DR-TB drug options and an important drug in children aged 3–6 years and XDR-TB.

Toxic drugs such as ethionamide (ETO), para-aminosalicylic acid (PAS) and amikacin (AM) may still be used but are poorly tolerated, whereas meropenem has to be given as intravenous infusion (IVI) twice a day for 6 months.

## Rifampicin-resistant tuberculosis regimens for adults, adolescents and children aged ≥ 6 years and weighing ≥ 16 kg

This section discusses the content of Table 1 in more detail.

**TABLE 1:** Updated drug-resistant tuberculosis regimens for adults and adolescents.

Resistance pattern	Other criteria	Intensive phase	Continuation phase
**RR/MDR-TB regimens FLQ-sensitive**			
Rif-resistant/FLQ sensitive/no more than one INH mutation	< 1-month exposure to second-line TB drugs in past Uncomplicated TB	4–6 months BDQ (6 M) + LZD (2 M) + LFX + hINH (4–6 M) + CFZ + EMB + PZA	5 months LFX + CFZ + EMB + PZA
Rif-resistant/FLQ sensitive With single or dual INH mutation	> 1-month exposure to second-line TB drugs in past Complicated TB (EPTB or extensive lung disease)	6–8 months BDQ + LZD + LFX + TZD + CFZ	12 months LFX + TZD + CFZ
**FLQ-resistant RR-TB regimens**			
Rif-resistant and FLQ-resistant	Use when highly suspicious of FLQ-resistant TB (e.g. contact history)	6–8 months BDQ + LZD + DLM + TZD + CFZ	12 months LZD + TZD + CFZ + (DLM or BDQ)
**Rescue regimen**			
Rif-resistant and any of BDQ/CFZ or LZD-resistant	Failure on any RR-TB regimen Where resistance to BDQ/LZD or CFZ is suspected	Consult NCAC	Consult NCAC
**Central nervous system RR-TB regimen**			
Rif-resistant (adjust regimen according to resistance pattern)	Confirmed or highly suspicious TB of meningitis	12 months BDQ (6 M) + LZD + LFX + CFZ + TZD + DLM + ETO (if Kat G) + hINH (if *inhA*) + PZA	6 months LFX + CFZ + TZD + DLM + (ETO or hINH or PZA)

BDQ, bedaquiline; LZD, linezolid; LFX, levofloxacin; CFZ, clofazamine; TZD, terizidone; DLM, delamanid; EMB, ethambutol; ETO, ethionamide; hINH, high-dose isoniazid; PZA, pyrazinamide; EPTB, extrapulmonary tuberculosis; FLQ, fluoroquinolones; INH, isoniazid; NCAC, National Clinical Advisory Committee; RR/MDR-TB, rifampicin-resistant/multidrug tuberculosis.

### The multidrug-resistant tuberculosis short regimen for uncomplicated rifampicin-resistant tuberculosis adults, adolescents and children aged ≥ 6 years (for 9–11 months)

The MDR-TB short regimen is an option for any newly diagnosed RR-TB patient, who has had no previous exposure to second-line drugs, has no more than one INH mutation and is still relatively well clinically. It is made up of seven medicines:

- bedaquiline as a 6-month course, but can be extended to 9 months in late converters
- linezolid for 2 months
- isoniazid for 4–6 months, which is stopped at 4 months if the acid-fast bacillus (AFB) is negative, followed by LFX, CFZ, PZA and EMB for 5 months.

Most patients will be on this regimen for 9 months, and those with late smear conversion may need 11 months to complete the regimen. We expect patients to be smear- and culture-negative at the end of 4 months or this regimen may have failed.

### The long basic multidrug-resistant tuberculosis regimen for complicated and re-treatment rifampicin-resistant tuberculosis adults, adolescents and children aged ≥ 6 years (for 18–20 months)

Any patient who is FLQ sensitive and does not qualify for short regimen will receive the basic long RR-TB regimen. This includes patients who have MDR-TB with dual mutation, patients who are injectable-resistant, patients with previous exposure to DR-TB drugs for > 1 month and complicated patients (e.g. extrapulmonary tuberculosis [EPTB] or extensive lung disease).

This regimen is longer but simpler. The five key drugs (Groups A and B) – BDQ, LFX, LZD, CFZ and terizidone (TZD) – are given for 6 months, followed by a continuation phase of 12 months with TZD, CFZ and LFX.

### The fluoroquinolones-resistant long rifampicin-resistant tuberculosis regimen

This regimen contains the biggest change from the interim guideline and is for patients with FLQ-resistant DR-TB (both XDR and pre-XDR FLQ-resistant). Owing to the addition of DLM, this regimen has been simplified dramatically and the duration is usually for no more than 18–20 months.

The regimen is similar to the MDR-TB long regimen, with LFX replaced by DLM. This is followed by a 12-month continuation phase of CFZ and TZD combined with one or two of LZD, BDQ or DLM. Patients with extensive disease would have four drugs in the continuation phase.

### The central nervous system rifampicin-resistant tuberculosis long regimen

One of the great challenges of some of our newer drugs, such as BDQ, is that they do not cross the blood-brain barrier very well. The regimen for meningitis TB is therefore the most complicated one, with inclusion of many Group C drugs with good cerebrospinal fluid (CSF) penetration.

The intensive phase has been increased to 12 months and has the basic long MDR-TB regimen (depending on LPA results) but also DLM, PZA and HHINH (if a patient has *inhA* mutation only) or ETO (if a patient has *katG* mutation only). A continuation phase of 4–5 drugs is recommended.

### The rifampicin-resistant tuberculosis rescue regimen

We already have patients with resistance to our new advanced drugs, including incidences of BDQ, CFZ and LZD resistance. These are key drugs to our regimens and any patient with suspected or proven resistance to any of these three will have a specific rescue regimen designed, which could include drugs such as DLM, mereponem, ETO, PAS and high-dose MOX. These regimens are designed by experts after having consultation with the National Clinical Advisory Committee (NCAC) at centres of excellence. It is imperative to request an extended-DST (eDST) through the National Health Laboratory Service (NHLS) on any patient where resistance to these drugs is suspected. The clinical reference guide^1^ outlines the steps of requesting an eDST at your own facility.

## Human immunodeficiency virus and rifampicin-resistant tuberculosis co-infection: Management of antiretrovirals of patients on rifampicin-resistant tuberculosis treatment

Being HIV-positive increases the chances of latent TB activation; conversely, TB leads to progression in HIV infection. Around 60% – 70% of patients with DR-TB can also have HIV infection, and this co-infection adds to the challenge of managing DR-TB successfully. This section discusses the content of Table 2 in more detail.

**TABLE 2:** Antiretroviral regimens for patients on drug-resistant tuberculosis treatment.

Criteria/regimen prior to DR-TB treatment	Regimen choice at start of DR-TB treatment	Regimen choice when BDQ/LZD completed	Proviso
New patient – never exposed to ARV, ≥ 10 years old and ≥ 35 kg	TDF/3TC/DTG as fixed combination	TDF/3TC/DTG as fixed combination	Ensure there is adequate creatinine clearance (for TDF use). Use appropriate formula for eGFR/creatinine clearance calculation according to age
Patient defaulted on 1TFE	TDF/FTC/LPV/r	AZT/3TC/DTG (second line). Change once HB ≥ 10 g/dL and LZD completed	
Patient defaulted on AZT/3TC/LPV/r (second line)	TDF/FTC/LPV/r	AZT/3TC/LPV/r (second line). Change once HB ≥ 10 g/dL and LZD completed	
Patient already on 1TFE: VL LDL	TDF/FTC/DTG	TDF/FTC/DTG	VL result in last 3–6 months
Patient already on 1TFE: VL > 1000	TDF/FTC/LPV/r	AZT/3TC/DTG (second line). Change once HB > 10 g/dL and LZD completed	Ensure there is adequate creatinine clearance (for TDF use)
Patient already on AZT/3TC/LPV/r	TDF/FTC/LPV/r	AZT/3TC/LPV/r (second line). Change once HB > 10 g/dL and LZD completed	Ensure there is adequate creatinine clearance (for TDF use)

Note: For more details on the regimens for children and patients on ABC, see the clinical reference guide.^1^

ARV, antiretroviral; AZT, zidovudine; FTC, emtricitabine; DR-TB, drug-resistant tuberculosis; VL, viral load; TDF, tenofovir; 3TC, lamivudine; DTG, dolutegravir; HB, haemoglobin; LPV/r, lopinavir/ritonavir; BDQ, bedaquiline; LZD, linezolid; eGFR, estimated glomerular filtration rate; VL LDL, viral load lower than detectable; 1TFE, first line with Tenofovir/Emitracitabine and Efavirenz.

*Drug interactions*: Efavirenz (EFV) and BDQ in combination are contraindicated because of potential lengthening of QT interval, and the combination of zidovudine (AZT) and LZD is contraindicated because of increased risk of bone marrow suppression.

Fortunately, the new antiretroviral (ARV) guidelines^3^ include dolutegravir (DTG) as the first-line option, and although DTG interacts with rifampicin (requiring a doubling of dose), it is safe to use and needs no adjustment with our DR-TB regimens. Although DTG is a very robust drug with a high genetic barrier, it needs to be given with two fully functional nucleoside reverse-transcriptase inhibitors (NRTIs). We cannot, therefore, simply switch patients already on older regimens to DTG unless viral suppression is done.

### Initiating the newly diagnosed human immunodeficiency virus-positive patients on rifampicin-resistant tuberculosis treatment

#### Initiating new patients with no previous antiretroviral exposure is straightforward

*Adults and adolescents aged > 10 years and weighing > 35 kg:* Tenofovir (TDF)/lamivudine (3TC) and DTG (TLD) in a fixed dose combination is the ideal choice. The challenge with DTG is the small possible risk of increased neural tube defects in mothers who conceive whilst on DTG. In the DR-TB programme, we advise patients not to fall pregnant as both the DR-TB drugs and DR-TB disease pose a risk to the unborn child. Women must be counselled about the risk of DTG and sign the informed consent form. They need to understand that once the TB treatment is completed and they wish to fall pregnant, they must consult their doctor about ARV options. Contraceptive choices must be made available.

*Children weighing 20–35 kg and aged less than 10 years*: TDF is not an option and the preferred regimen is abacavir (ABC), 3TC and DTG.

### Modification of antiretroviral regimen in patient who has interrupted antiretrovirals

Patients who have defaulted treatment are usually restarted on the same regimen, but we have to make allowance for drug interactions with the DR-TB treatment.

*Patients on first line TDF/FTC/EFV (1TFE) before lost to follow-up (LTFU):* Switch to TDF/FTC and lopinavir/ritonavir (LPV/r) but as soon as LZD is completed and the haemoglobin (HB) ≥ 10 g/dL, the patient can be switched to the new second-line regimen: AZT, 3TC and DTG.

*Patients on second-line AZT/3TC/LPV/r before LTFU*: Switch to TDF/FTC and LPV/r, but once LZD is completed and HB ≥ 10 g/dL, the patient can be switched back to AZT, 3TC and LVP/r.

### Modification of antiretroviral regimens in patients already on antiretrovirals

For those patients already on ARVs, the modification to the antiretroviral therapy (ART) regimen will depend on the last viral load (VL), preferably taken in the last 3 months. Always check the VL and CD4 cell count at baseline.

*Patients on 1TFE and VL LDL*: These patients can be switched to TLD with the same caveats for women in child-bearing age as mentioned above.

*Patients on 1TFE and VL > 1000*: However, if VL is > 1000, such patients can be prepared for second-line treatment.

As we cannot use AZT, start patient on TDF, emtricitabine (FTC) and LPV/r; but as soon as LZD is stopped and HB ≥ 10 g/dL, the patient can be switched to the new second-line ARV regimen: AZT, 3TC and DTG.

*Patients on second-line AZT/3TC/LPV/r*: Patients already on second-line regimen have to be switched to TDF, FTC and LPV/r and, as soon as possible, the TDF is to be switched back to AZT. Do not use the new second-line regimen.

The guidelines recommend that patients with VL 50–1000 are to be discussed with NCAC.

## Conclusion

It is not possible to cover all of the information contained in the clinical reference guide^1^ which also contains sections on the diagnosis and management of children younger than 6 years, the principles of treatment in pregnancy and breastfeeding, managing adverse events (particularly anaemia, peripheral neuropathy, increased QT interval and hepatotoxicity) and post-exposure management. Primary care clinicians are advised to review these additional sections to ensure that their knowledge remains up to date. It is believed that this continuing professional development (CPD) article would be useful when providing decentralised care to patients diagnosed with RR-TB.
